# Comparison of clinical outcomes with proximal femoral nail anti-rotation versus bipolar hemiarthroplasty for the treatment of elderly unstable comminuted intertrochanteric fractures

**DOI:** 10.1186/s12891-022-05583-4

**Published:** 2022-07-01

**Authors:** Qi-Chun Song, Sha-Jie Dang, Yan Zhao, Ling Wei, Da-Peng Duan, Wen-Bo Wei

**Affiliations:** 1 First Department of Orthopaedics, The Second Affiliated Hospital of Xi’an Jiaotong University, Xi’an, 710004 Shaanxi China; 2 Department of Anesthesia, Shaanxi Provincial Cancer Hospital, Xi’an, 710061 Shaanxi China; 3 The Key Laboratory of Biomedical Information Engineering of Ministry of Education, School of Life Science and Technology, Xi’an Jiaotong University, Xi’an, 710049 Shaanxi China; 4 Department of Pain, Yangling Demonstration Zone Hospital, Yangling, 712100 Shaanxi China; 5 Department of Orthopedics, Shaanxi Provincial People’s Hospital, Xi’an, 710068 Shaanxi China

**Keywords:** Intertrochanteric fracture, Elderly, Unstable, Proximal femoral nail antirotation, Bipolar hemiarthroplasty

## Abstract

**Background:**

Although proximal femoral nail anti-rotation (PFNA) and bipolar hemiarthroplasty (BHA) are selected by most of the orthopaedic surgeons for elderly intertrochanteric fractures (ITFs) patients, there is still no consensus on the superiority of PFNA and BPH for the elderly with unstable comminuted ITFs. The study aims to compare the curative effects of PFNA and cementless BHA on unstable comminuted ITFs in the elderly.

**Methods:**

From January 2012 to December 2016, we retrospectively reviewed 62 ITFs patients up to the inclusion and exclusion criteria in the study. Depending on the type of surgery, the patients were divided into two groups: Group BHA (*n*= 30) and Group PFNA (*n* = 32). The ITFs were classified according to Evans-Jensen. Hospitalization time, operation time, bleeding loss, weight bearing duration, Harris hip scores, 10-m walking speed, gait and postoperative complications were compared between the two groups.

**Results:**

There was no significant difference between the groups in hospital stay (*P* > 0.05). The BHA group trended to have a shorter operation time and a larger volume of blood loss (*P* < 0.01).The weight bearing duration was shorter in the BHA group than the PFNA group (*P* < 0.05).The Harris hip score was higher, the 10-m walking speed was faster and the gait was better in group BHA than group PFNA at three months postoperatively (*P* < 0.05), but there was no significant difference between the two groups at 6 and 12 months postoperatively (*P* > 0.05). There was no significant difference in postoperative complications between the two groups (*P* > 0.05).

**Conclusion:**

The BHA allows an earlier return to weight-bearing activity, but ultimately has the same effective treatments as the PFNA for the elderly with unstable comminuted ITFs.

## Introduction

Femoral intertrochanteric fracture (ITFs), in particular, unstable comminuted fractures, is a common hip fracture that occurs in the elderly [1, 2]. The incidence is gradually increasing with the recent growth of the elderly population [3]. Due to the higher mean age, poor quality of bone mass, and a large number of underlying diseases, patients with this fracture have high rates of complications and mortality [4, 5]. To reduce disability and mortality rates, the early surgical procedure has become the general consensus for the ITFs treatment [6]. The key point of the surgical treatment is stable fixation and early weight-bearing. Considering the elderly’s age, underlying comorbidities, quality of bone, and type of fracture, different from the various operation methods for young ITFs patients, proximal femoral nail antirotation (PFNA) was superior to hemiarthroplasty (BHA) in the elderly according to the operative statistics, but there were no significant differences in functional outcome [7]. PFNA exactly has many advantages in terms of the small surgical wound, easy implant insertion, and stable fixation [8], but failure to achieve early weight-bearing [9]. BHA, which is advantageous in terms of operation time and allowing early weight-bearing, has been suggested as another surgical option for elderly patients [10]. However, there is currently no consensus for elderly patients with unstable comminuted ITFs [11]. In this retrospective study, we compared the efficacy and complications of PFNA and BHA, and want to address which is optimal for treat unstable comminuted ITFs?PFNA or BHA.

## Methods

### Study design

We retrospectively reviewed the medical records of 62 patients up to the inclusion and exclusion criteria, who were treated at the department of orthopedic surgery, Shannxi Provincial People’s Hospital between January 2012 and December 2016. This study was approved by the clinical research ethics committee of Shannxi Provincial People’s Hospital (No. 2017–018). This study followed the Good Clinical Practice guidelines and the guidelines of the Helsinki Declaration. The study included 32 cases underwent PFNA (Group PFNA) and 30 cases underwent BHA (Group BHA).

### Patients

Patients (aged 65~98 years old) undergoing PFNA or BHA operation as ITFs were screened in this study. Inclusion criteria were 1) ≥65 years old; 2) patients with a fracture that occurred after trauma; 3) Unstable comminuted ITFs according to Evans-Jensen classification (Evans-Jensen III, IV and V);4) T-scores of health femoral head or lumbar bone mineral density (BMD) were less than -2.5. Exclusion criteria were 1) pathologic fractures; 2) fractures associated with polytrauma; 3) immobility or walking difficulties before fracture; 4) Patients who are unable to operate due to mental or organ dysfunction, and 5) patients who are lost to follow-up.

### Procedures

Operations were performed under spinal anaesthesia or general anaesthesia.

### PFNA group

**Fig. 1:**
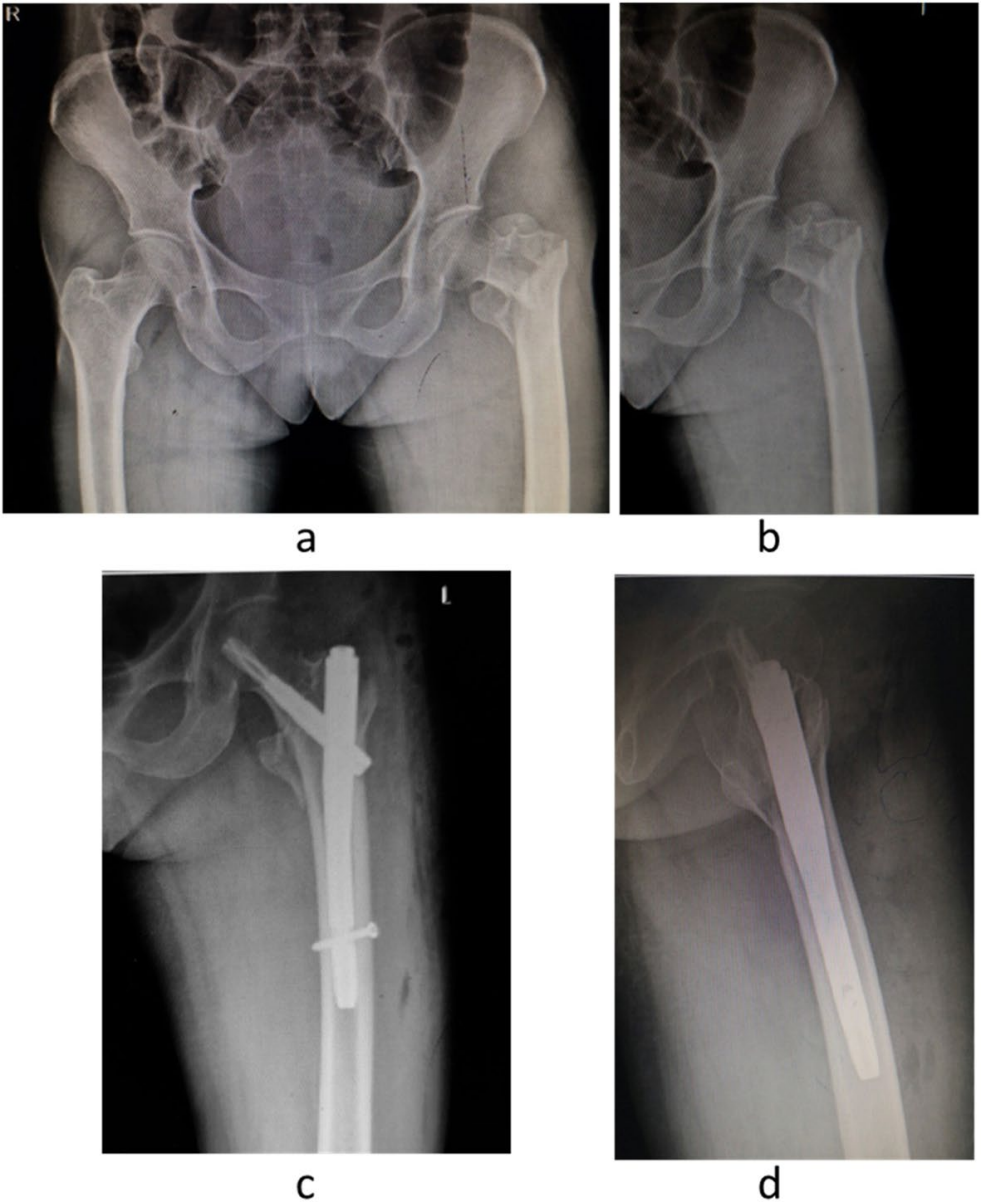
Anteroposterior radiograph showing an unstable intertrochanteric fracture of left hip in a 76-year-old female patient who fell at home (**a, b**). Anteroposterior pelvis and lateral femoral examination after the operation showed good fracture alignment and satisfactory fixation (**c, d**)

The patient was lying on the traction bed in supine position. The fracture was reset under C-arm fluoroscopy guidance by a standard program. After satisfactory reduction, a straight incision 3- to 5- cm long was made from the top of the greater trochanter toward the proximal side. A rhombus-shaped awl was used to drill a hole at the front and middle 1/3 between the tip of the greater trochanter. Then the proximal femoral nail was inserted, which was matched with the femoral bone marrow cavity. Under C-arm fluoroscopy, the column screw was knocked in until its tip as close as 5 mm to the subchondral bone. We fix the locking bolt and the end cap, then close the wound in layers. The PFNA material was provided by the WeiGao Company (Weihai, China) and the DaBo Company (Xiamen, China) (Fig. 1).

### BHA group

**Fig. 2:**
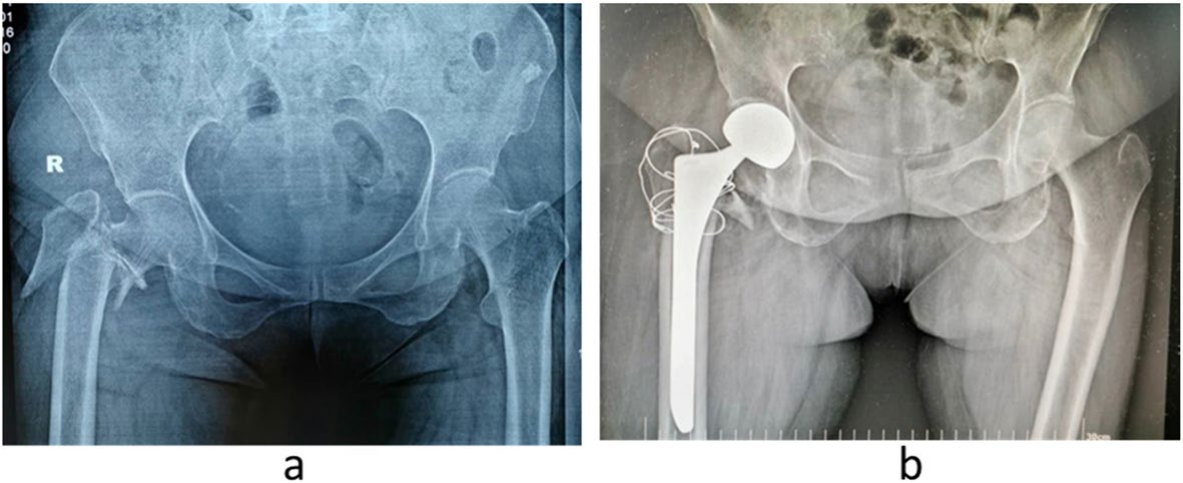
**a** Anteroposterior radiograph showing an unstable intertrochanteric fracture of right hip in a 82-year-old male patient who fell at home. **b** Radiograph one year after hemiarthroplasty

The patient was in a lateral decubitus position. Using posterolateral invasive approach, layer- by-layer incisions were made to expose the fracture site. We cut the joint capsule, performed femoral neck osteotomy, and expanded the medullary cavity by use of medullary cavity burs. A suitable biological long-stem femoral prosthesis was selected according to the preoperative X-ray measurement and the actual intraoperative status of the medullary cavity. The anteversion angle of the femoral stem was maintained at 15°-20°, the femoral head model was inserted, and the hip joint was reduced. Displaced greater trochanter fracture fragments were fixed by wire as a ‘8’ shape. The stability of the reduction was tested after ensuring the absence of dislocation. After satisfactory results were obtained, the corresponding femoral prosthesis and the femoral bipolar head were implanted. We sutured the joint capsule, reconstructed the external rotator muscles, and stitched the wound. Long-stem biotype artificial joint was provided by the Chunli Company (Beijing, China) and the Link Company (Germany) (Fig. 2).

All the patients used antibiotic prophylaxis within 30 min before incision and the first 24 h postoperatively. Low molecular weight heparin or rivaroxaban was used within 30 days after the operation.

### Outcomes

The patients’ medical information was obtained from the patients’ clinical history and Medical Records Department. The patients’ general conditions were ranked by the American Society of Anesthesiologists (ASA) grading. The fractures were classified according to association for the study of Evans-Jensen classification together by two surgeons through radiographs. We recorded the time when the patient began to weight bearing after operation. Weight-bearing duration was determined as the patient being able to walk continuously for 3 min or more, with a walking distance no fewer than 30 steps, after walking-aid instrument removal. When patients visit, anteroposterior and lateral radiograph with a standard questionnaire was performed, and hip function was evaluated according to 10-m walking speed and gait test, and the Harris hip score included in the standard questionnaire. During the 3, 6, 12 months follow-up, after the walking-aid instrument was removed and if no deformation was found in the fracture site, 10-m walking speed and gait were measured by a nurse with professional training. Three trials were conducted in succession and the average time was taken. 1.5 Statistical analysis.

The statistical analysis was performed with SPSS 24.0 for Windows (SPSS, Inc., IBM). Measured data were tested for normal distribution and the homogeneity of variance. Numeric variables were expressed as Mean ± SD and analyzed by Independent-Samples T-test. Categorical data were expressed by N (%) and were analyzed with the χ^2^ test. The value of *p* < 0.05 was taken as a significant difference.

## Results

### General information

A total of 245 ITFs patients were reviewed, 42 patients were excluded for there were 3 pathologic fractures, 10 with walking difficulties before fracture, 29 fractures associated with polytrauma, and 129 patients were excluded because of low grade Evans-Jensen classification (I and II), high bone density(T≥-2.5), walking difficulties before fracture or surgical contraindication due to mental or organ dysfunction, and 12 patients were lost due to failed followed up. Finally, 62 patients were followed up successfully and then included in the study.

There was no significant difference in the gender, age, the American Society of Anesthesiologists (ASA) grading, the time from injury to operation, and Evans-Jensen classification between the two groups (*P* > 0.05). As for the combining metabolic disease, the number of diseases per patient in the BHA group was higher than that in PFNA group(*P* = 0.039, T-test). There were no differences in cardiovascular disease, diabetes, chronic pulmonary disease, cerebrovascular disease, neurological disease, and hypertension between the two groups (Table 1).

**Table 1:** The comparison of baseline characteristics between BHA and PFNA

Characteristics		BHA	PFNA	χ^2^/*t*	*P*
Cases (n)		30	32	-	-
Gender (M/F)		9/21	5/27	1.830	0.230
Age(x ± s, years)		81.0 ± 9.1	79.9 ± 6.1	0.541	0.591
ASA grading	3	11	16	2.264	0.132
	4	21	14		
Evans-Jensen classification	3	5	5	2.246	0.884
	4	8	7		
	5	17	20		
Time from injury to operation(d)		5.06 ± 2.17	4.70 ± 1.17	0.766	0.447
Mean N of diseases per patient		2.47 ± 0.567	2.07 ± 0.907	2.107	0.039
Hypertension		15	11	0.663	0.416
Cardiovascular disease		19	12	2.325	0.127
Diabetes		8	8	0.022	0.881
Chronic pulmonary disease		16	14	0.069	0.793
Cerebrovascular disease		15	15	0.061	0.806
Neurological disease		6	2	2.246	0.156

*Notes*: Numeric data were expressed as Mean ± SD and analyzed by Independent-Samples T-test. Categorical data were expressed by the number of patients (%) and were analyzed with the χ.^2^ test. *P < 0.05, Group BHA vs Group PFNA

*Abbreviations*: *ASA* American Society of Anesthesiologists, *PFNA* proximal femoral nail antirotation; *BHA* bipolar hemiarthroplasty

### Comparison of hospitalization and operation conditions

There was no significant difference between the groups in hospital stay. However, the operative statistics including the operating time (*P* < 0.001, T-test) and the bleeding volume (*P* < 0.001, T-test) were quite different. The BHA group trended to have a shorter operation time (94.38 min vs. 125.67 min), and a larger volume of blood loss (335.31 ml vs. 153.33 ml) (Table 2).

**Table 2:** The comparison of hospitalisation and operation between BHA and PFNA

Characteristics	BHA(*n* = 30)	PFNA(*n* = 32)	*t*	*P*
Operating time(min)	94.38 ± 20.94	125.67 ± 33.49	-4.441	< 0.001
Bleeding volume(ml)	335.31 ± 90.87	153.33 ± 59.96	9.241	< 0.001
Hospital stay(d)	16.63 ± 3.64	17.13 ± 2.92	-0.604	0.548
weight bearing(d)	11.15 ± 1.36	18.42 ± 1. 75	-5.319	0.014

*Notes*: Numeric data were expressed as Mean ± SD and analyzed by Independent-Samples T-test. *P < 0.05, Group BHA vs Group PFNA

*Abbreviations*: *PFNA* proximal femoral nail antirotation, *BHA* bipolar hemiarthroplasty

### Comparison of functional outcomes

After the operation, the weight bearing duration was shorter in the BHA group than the PFNA group (*P* < 0.05, Table 2). As for the functional aspects evaluated by the Harris hip score at 3 months follow-up, the BHA group scored (68.91 ± 8.15) better than the PFNA group (73.20 ± 6.56) (*P* < *0.05*). However, there was no significant difference between the groups at 6 and 12 months follow up.

10-m walking speed in the BHA group was faster than in the PFNA group at 3 months of the post-operation (*P* < 0.05), but there was no significant difference between the groups at 6 and 12 months. At the same time, the number of people in the BHA group with normal gait was higher than that in the PFNA group and the number of people in the PFNA group with severe lameness was higher than that in the BHA group at 3 months postoperatively (*P* < 0.05), but there was no significant difference between the two groups at 6 and 12 months postoperatively (Table 3).

**Table 3:** The comparison of Harris hip Score, 10-m walking speed and gait between BHA and PFNA

		3 months of the post-operation			6 months of the post-operation			12 months of the post-operation		
Harris hip Score	BHA group (*n* = 30)	73.20 ± 6.56			78.15 ± 9.46			79.95 ± 7.19		
	PFNA group (*n* = 32)	68.91 ± 8.15			77.56 ± 8.79			78.39 ± 8.27		
	*P* value	0.027			0.799			0.432		
10-m walking speed	BHA group (*n* = 30)	0.91 ± 0.21			1.14 ± 0.40			1.43 ± 0.17		
	PFNA group (*n* = 32)	0.64 ± 0.18			0.95 ± 0.62			1.38 ± 0.21		
	*P* value	0.021			0.684			0.749		
Gait		A	B	C	A	B	C	A	B	C
	BHA group (*n* = 30)	8	17	5	17	10	3	23	6	1
	PFNA group (*n* = 32)	3	15	14	15	12	5	22	8	2
	*P* value	0.036			0.689			0.749		

*Notes*: Numeric data were expressed as Mean ± SD and analyzed by Independent-Samples T-test. Categorical data were expressed by the number of patients and were analyzed with the χ.^2^ test. *P < 0.05, Group BHA vs Group PFNA

*Abbreviations*: *PFNA* proximal femoral nail antirotation, *BHA* bipolar hemiarthroplasty, A: Normal gait; B: Mild to moderate lameness; C: Severe lameness

### Postoperative complications

Postoperative complications in the PFNA group include infection in three cases, symptomatic DVT in one case, cutout in three cases, and new fracture around the implant in three cases; in the BHA group include infection in two cases, symptomatic DVT in five cases and new fracture around the implant in three cases. There was no significant difference between the groups (Table 4).

**Table 4:** Postoperative complications of BHA and PFNA

Characteristics	BHA(*n* = 30)	PFNA(*n* = 32)	χ^2^	*P*
Infection	2	3	0.294	0.588
Symptomatic DVT	5	1	2.676	0.102
Cutout	0	3	3.363	0.067
New fracture around the implant	3	3	0.120	0.729

*Notes*: Categorical data were expressed by the number of patients (%) and were analyzed with the χ.^2^ test. *P < 0.05, Group BHA vs Group PFNA

*Abbreviations*: *PFNA* proximal femoral nail antirotation, *BHA* bipolar hemiarthroplasty

## Discussion

Due to the aging of the population, the number of elderly patients with unstable comminuted ITFs is increasing gradually. These ITFs patients have difficulties to return to prefracture function levels and display poor treatment results because of low bone quality, additional morbidities, and mobilization problems [8]. An ideal surgical technique for elderly patients with unstable comminuted ITFs should be less trauma and postoperative complications [12]. However, it is still unclear whether BPH or PFNA is the better choice for these patients. Thus, our study was initiated to compare the PFNA and BHA groups and to help orthopedic surgeons to choose a suitable implant to fix ITFs in elderly patients with unstable comminuted ITFs.

As a minimally invasive procedure, PFNA offers the advantages of micro-trauma, minimal bleeding, and short operation time [13]. PFNA nails not only reduces movement, sliding compression but also increases the anti-rotation screw, which significantly enhances the anti-rotation, anti-compression, and anti-tension abilities of the fracture end, increases the stability of the fracture end [14]. Thus, PFNA is particularly suitable for ITFs, which minimizes the risk of medical complications. However, for elderly intertrochanteric fracture patients with Evans-Jensen type III or above, the ITFs caused the loss important mechanical effects, such as support of the femoral neck, anti-rotation and anti- introversion. Intraoperative fracture reduction is difficult, and in femoral necks with serious osteoporosis, screw loosening and cutting are likely to occur.

In our results, the PFNA group has less blood loss, but longer operating time than BHA, which is different from the previous literature [13]. The patients in our study have unstable comminuted fractures, so intraoperative closed traction reductions take longer time. Besides, a lot of intraoperative fluoroscopies are used, to avoid intraoperative complications, such as the internal fixation point explosion, cut-off of needles from the medial wall of femur, the separation of the end of fracture, etc. Although PFNA has been selected by most surgeons for elderly ITFs patients [15–17], failures of PFNA have also been reported due to extensive comminution, osteoporosis, or long bedridden duration [17]. PFNA complications include cutout of the femoral screw, breakage of the nail, split of the lateral cortex of the proximal femur, and fracture of the femoral shaft [18]. In our results, the femoral heads of two patients were cutout and the femoral shafts of three patients were splited by the implants in PFNA group, which may be related to comminution fracture and osteoporosis.

BHA, which is advantageous in terms of less operation time and allowing early weight-bearing, was first used in 1978 and subsequently used by other surgeons for ITFs treatment with satisfying results [19], has been suggested as an alternative method for elderly ITFs patients [7, 20]. BHA is recommended as a prior treatment for ITFs with poor stability in the elderly with severe osteoporosis, poor prognosis after internal fixation, and a short life expectancy [21]. This study reaveals the weight bearing was earlier in the BHA group than the PFNA group, and Harris score, 10-m walking speed and gait in the BHA group was superior to the PFNA group at 3 months, which indicates that the use of long-stem cementless prosthesis in BHA can begin functional exercise earlier and obtain preeminent effective treatments for the elderly with unstable comminuted ITFs, in spite of accompanying with more blood loss. Howerver, the superiority of BHA was regressive at 6 and 12 months. Early ground movement may be related to the function of BHA to more effectively immobilize shattered bones around it.

In comparison, BHA can quickly restore hip function; it is mainly used to treat femoral neck fractures in the elderly, including unstable intertrochanteric fractures and failure of intertrochanteric fracture fixation [22]. Haentjens et al. [21] reviewed the relevant literature and showed that intertrochanteric comminuted fracture patients with severe osteoporosis may benefit from BHA. There is also controversial regarding the choice of cemented and cementless prostheses. Some studies have reported that with the improvement and development of implant design, materials and insertion techniques, the use of cementless prosthesis for artificial femoral replacement in elderly patients with unstable ITFs can achieve better results compared with cemented prostheses [23, 24]. In this study, the BHA group was treated with cementless acquiring considerable efficacy.

There were several limitations in our study. Firstly, small sample size of clinical cases and a retrospective study rather than prospective study, further study is needed in therapeutic regime. Secondly, quite a few cases were excluded that can cause bias and affect the reliability of the results. Lastly, baseline data for the two groups were not very consistent, such as the number of medical diseases per patient and anti-osteoporosis medication, which may have contributed to inaccurate results.

## Conclusion

In conclusion, BHA and PFNA are two safe and effective fixation methods for treating the elderly with ITFs for it can obtain stable fracture fixation. The BHA allows an earlier return to weight-bearing activity and walking, but ultimately has the same effective treatments as the PFNA for the elderly with unstable comminuted ITFs. Clinicians should cautiously control surgical indications and choose the most effective internal implants that is reasonable to obtain the most satisfactory clinical curative effect.

## Data Availability

The authors will allow the sharing of participant data. The data will be available to anyone who wishes to access them for any purpose. The data will be accessible from immediately the following publication to 6 months after publication, and contact should be made via the first author by email.

## Abbreviations

PFNA: Proximal femoral nail anti-rotation
BHA: Bipolar hemiarthroplasty
ITFs: Intertrochanteric fractures
ASA: American Society of Anesthesiologists
